# Benign tumor mimicking cancer in breast: a case report

**DOI:** 10.3389/fonc.2025.1602732

**Published:** 2025-09-29

**Authors:** Tingting Ding, Yu Zheng, Jianyong Zhang, Yunsong Peng

**Affiliations:** ^1^ Department of Pathology, Affiliated Hospital of Guizhou Medical University, Guiyang, Guizhou, China; ^2^ Department of Breast Surgery, Affiliated Hospital of Guizhou Medical University, Guiyang, China; ^3^ Department of Vascular and Thyroid Surgery, Guizhou Provincial People’s Hospital, Guiyang, Guizhou, China; ^4^ Department of Medical Imaging, Guizhou Provincial People’s Hospital, Guiyang, Guizhou, China

**Keywords:** breast granular cell tumor, breast cancer, misdiagnosis, radiology, histopathology

## Abstract

Breast granular cell tumor (BGCT) is a rare neoplasm that typically presents as a benign lesion but is frequently misdiagnosed as breast cancer prior to biopsy. Herein, we report a case of BGCT that was initially suspected to be breast cancer based on preoperative physical examination and imaging findings. A 39-year-old Asian woman presented with a firm and painless mass in the right breast. Color Doppler ultrasonography revealed a 15 mm × 15 mm × 14 mm nodule in the upper inner quadrant of the right breast without obvious blood flow signal. On magnetic resonance imaging (MRI), dynamic contrast-enhanced sequences demonstrated homogeneous enhancement. Both ultrasonography and MRI reported the lesion as the Breast Imaging Reporting and Data System (BI-RADS) Category 4B. Based on these findings, the patient was clinically suspected to be an early breast cancer. A surgical plan was formulated, beginning with an excisional frozen section with negative margins and proceeding to breast-conserving surgery if necessary. Frozen section analysis confirmed the presence of a tumor but could not determine whether the lesion was benign, malignant, or borderline. Histopathological examination with hematoxylin-eosin staining and immunohistochemistry ultimately established the diagnosis of BGCT. Early and accurate diagnosis is crucial for developing appropriate treatment plans for breast neoplasms. Given the unique characteristics and rarity of these tumors, clinicians, radiologists and pathologists should remain vigilant and consider the possibility of BGCT in the differential diagnosis.

## 1 Introduction

Granular cell tumor (GCT) is a rare neoplasm (1), typically benign, with malignancy occurring in fewer than 1% of cases (2). It is currently widely believed to originate from Schwann cells of peripheral nerves (3). GCTs can arise in virtually any part of the body and may present as multicentric lesions (4). They most commonly occur in the head and neck region, with breast involvement being relatively rare, accounting for approximately 6–8% of all cases (5). A breast granular cell tumor (BGCT) prevalence of 1:1000 to 1:600 among breast malignancies has been widely reported (6). Notably, BGCT can coexist with breast carcinoma (6). Definitive diagnosis of BGCT relies on histopathological examination and immunohistochemistry (7). Complete surgical excision remains the most effective treatment strategy (1, 7). This case is of particular interest because BGCT can closely mimic invasive breast carcinoma on clinical and radiologic evaluation. Its rarity and overlapping features with malignancy may lead to misdiagnosis and potentially inappropriate treatment.

Clinically, when a breast lesion is suspected to be malignant, mammography, ultrasonography, and magnetic resonance imaging (MRI) are all recommended imaging modalities (8, 9). Additionally, several derivative imaging techniques have been developed to differentiate breast lesions, such as ultrasonography contrast imaging and digital breast tomosynthesis (10, 11). In Asia, ultrasonography is routinely used as the initial imaging modality for premenopausal patients with breast lesions. In cases of fatty breast tissue, mammography is also recommended. If physical examination or ultrasonography suggests a high likelihood of malignancy, mammography and MRI are directly added. Herein, we report a case of BGCT that mimicked carcinoma on ultrasonography and MRI. The diagnosis was confirmed by histological examination, and this case is presented to enhance readers’ understanding of BGCT.

## 2 Case presentation

**Figure 1:**
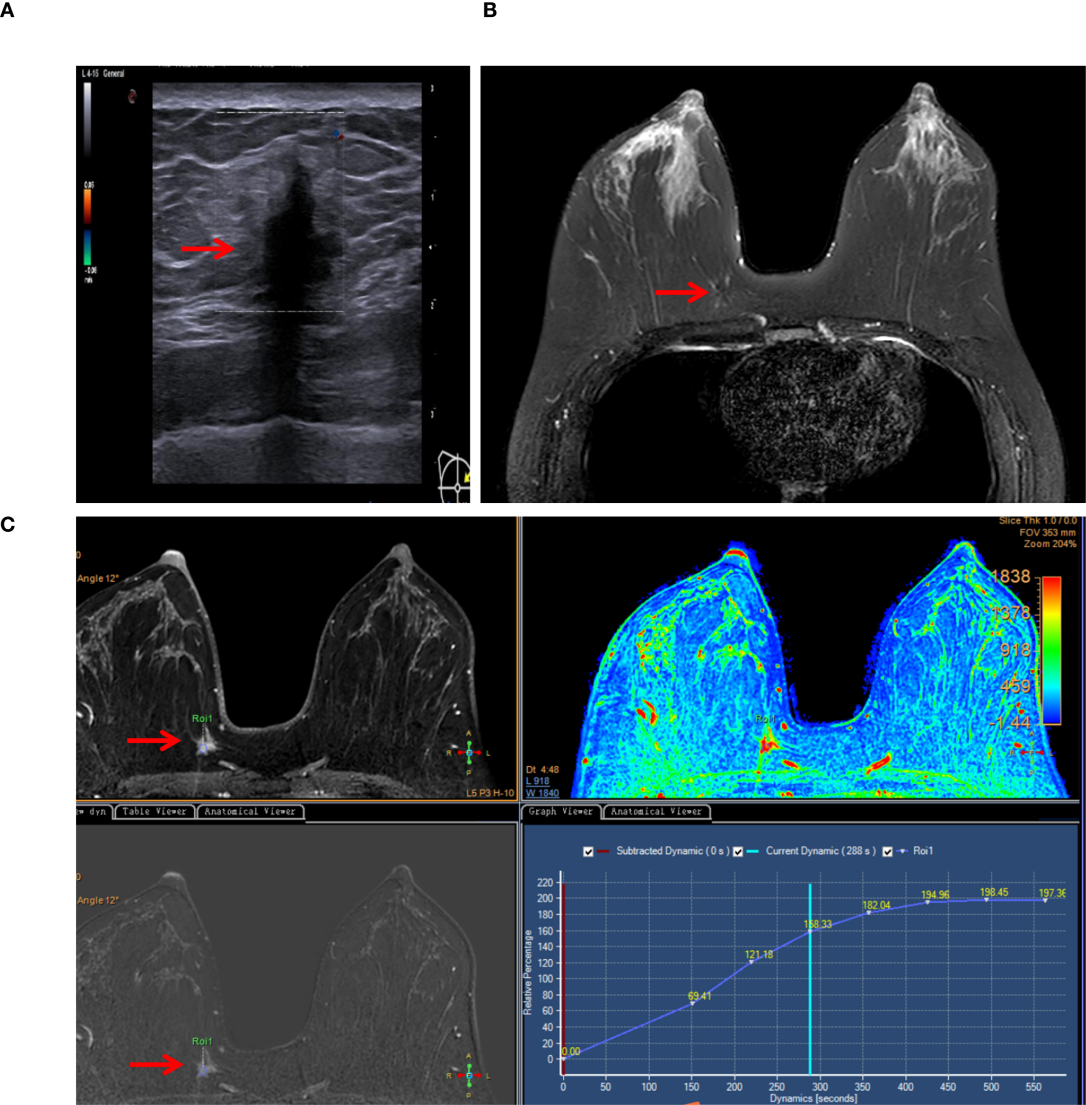
Typical imaging findings (lesion marked by arrows). **(A)** Color Doppler ultrasonography detected a noncapsular mass in the upper inner quadrant of the right breast (15×15×14 mm). **(B)** Breast MRI revealed a mass in the right breast (15×13×8 mm), exhibiting slightly high signal intensity on T2-weighted imaging (T2WI). **(C)** MRI dynamic contrast enhancement, silhouette image, Apparent diffusion coefficient (ADC) mapping (pseudo-color image), dynamic contrast enhancement and the time-intensity curve (TIC).

A 39-year-old woman presented to our department with a 10-day history of a right breast mass, initially detected during a routine health check-up. She had no known personal or family history of breast cancer. Physical examination revealed a palpable and firm and painless mass in the upper inner quadrant of the right breast, with unclear margins. No enlarged lymph nodes were palpable in the right axilla. Color Doppler ultrasonography of the right breast identified a hypoechoic nodule at the 2–3:00 position at the edge of the glandular tissue in the right breast (**Figure 1A**). The nodule measured approximately 15 mm × 15 mm × 14 mm, with an irregular shape, angulated and spiculated margins, uniform internal echoes, significant posterior acoustic shadowing, and no obvious blood flow signal. It was classified as the Breast Imaging Reporting and Data System (BI-RADS) Category 4B. Breast MRI showed a mass in the upper inner quadrant of the right breast, measuring approximately 15 mm × 13 mm × 8 mm. The mass exhibited low signal intensity on T1-weighted imaging (T1WI), slightly high signal intensity on T2-weighted imaging (T2WI) (**Figure 1B**), central low signal intensity, and slightly high signal intensity on diffusion-weighted imaging (DWI). Apparent diffusion coefficient (ADC) mapping revealed reduced signal intensity, and dynamic contrast enhancement demonstrated homogeneous enhancement. The time-intensity curve (TIC) exhibited a slow-rising pattern, and the lesion was classified as BI-RADS 4B (**Figure 1C**). Mammography was not performed due to the lesion’s small size, its location in the upper-inner quadrant of the right breast edge, and its proximity to the chest wall, which rendered mammography unsuitable for evaluation. Computed tomography (CT) of chest and abdominal ultrasonography showed no abnormalities. There was no evidence of axillary lymph node enlargement according to her imaging findings.

**Figure 2:**
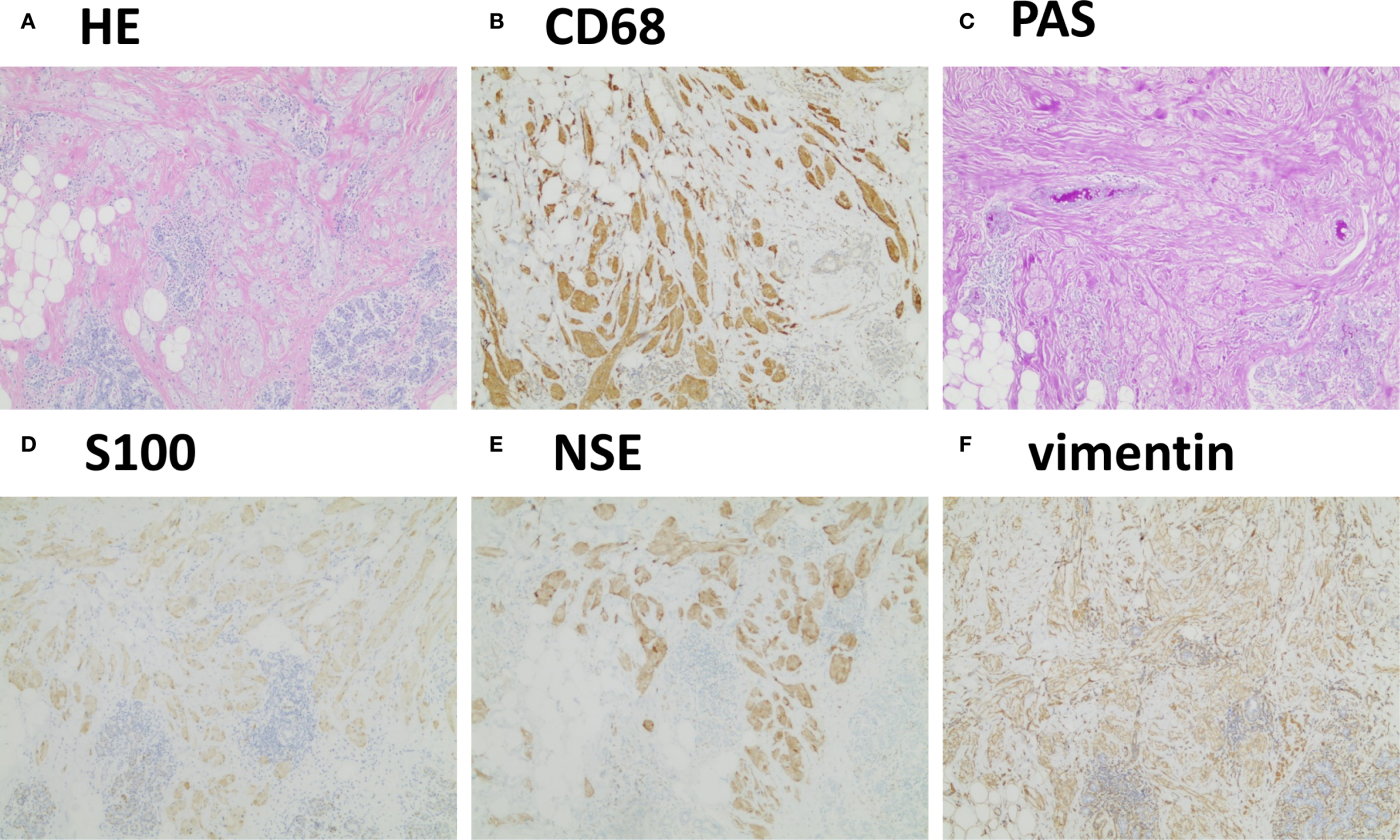
Typical histopathological findings. **(A)** Hematoxylin–eosin staining revealed disrupted normal breast tissue architecture with nests of atypical cells featuring abundant cytoplasm. **(B-F)** Immunohistochemistry showed positivity for CD68, PAS, S100, NSE, and vimentin (4×).

Given the suspicion of early breast cancer, a surgical plan was made to perform a wide excision first because of the infiltrative pattern of the lesion under general anesthesia for pathological investigations. If intraoperative frozen section analysis indicated malignancy, a breast-conserving surgery with radical resection would be considered. During surgery, frozen section analysis suggested that the lesion was likely a benign tumor, and that there were no tumor cells at the surgical margins. Postoperative histopathological examination with hematoxylin-eosin staining revealed disrupted normal breast tissue architecture with nests of large polygonal tumor cells with abundant eosinophilic granular cytoplasm infiltrating the surrounding breast parenchyma (**Figure 2A**), consistent with a tumor. Immunohistochemical staining results were as follows: S100 (+), CK (−), Vimentin (+), CD68 (+), CK7 (−), CK5/6 (−), PAS (+), NSE (+), Ki-67 (1%+), E-cadherin (+), P120 (+), Calretinin (+), α-Inhibin (+), SOX10 (+), P53 (wild-type), HER2 (0), GATA3 (−), ER (−), PR (−), GCDFP-15 (−), Mammaglobin (−), Desmin (−), SMA (−), PAX8 (−), P63 (−) and AR (−). Several of these markers are shown in **Figures 2B-F**. The final diagnosis was a benign BGCT.

## 3 Discussion

BGCTs typically present as firm, painless palpable masses (1). While imaging may occasionally reveal well-circumscribed lesions suggestive of benign pathology (4, 12), BGCTs often appear spiculated or poorly defined, mimicking the radiological features of breast carcinoma and thus are frequently misdiagnosed preoperatively (2, 13–16). These tumors are most commonly located in the upper inner quadrant, as in the case we report. This distribution is currently attributed to their origin from the intramammary branches of the supraclavicular nerve, whereas primary breast carcinomas more frequently arise in the upper outer quadrant (13). Surgeons, radiologists and pathologists should remain vigilant and consider these tumors as part of the differential diagnosis when evaluating breast masses.

Radiologic imaging has limited sensitivity in detecting BGCTs (17, 18). Ultrasonographically, benign BGCTs may display malignant features such as a solid, heterogeneous mass with indistinct margins and a high depth-to-width ratio (19), as demonstrated in our case. However, some cases may show benign characteristics with well-defined margins (4, 12). On mammography, BGCTs typically appear as small lesions (<3 cm), though lesions up to 6 cm have been reported (7). They may present as circumscribed masses or indistinct, spiculated lesions without calcification, further complicating differentiation from malignancy (20). MRI, including dynamic contrast-enhanced sequence, has limited sensitivity for diagnosing BGCTs but remains valuable for assessing lesion extent and multifocality (21, 22). Some studies report low-to-intermediate signal intensity on T1WI and a lack of hyperintensity on T2WI, consistent with our case. Although dynamic MRI kinetic curves and enhancement patterns may aid in distinguishing between benign and malignant lesions, the role of DWI and ADC values in assessing tumor aggressiveness remains controversial (23). To date, no specific imaging features have been definitively associated with BGCTs. Some authors reported homogeneous enhancement on T1WI images and ring-like enhancement on T2WI sequences. T1WI signals are typically low to intermediate, while T2WI signals can be variable (24). For breast masses in which malignancy cannot be excluded, we believe that contrast-enhanced ultrasonography (CEUS) serves as an important supplementary diagnostic tool (25). CEUS can further reveal the blood perfusion characteristics and dynamic changes of the lesion, thereby aiding in the evaluation of its nature, particularly for those classified as BI-RADS 4 based on Doppler ultrasonography findings.

Grossly, BGCTs appear as small, firm, grayish-white nodules with a dense cut surface. Microscopically, the tumor is characterized by aggregates of loosely infiltrating large round or polygonal cells with abundant eosinophilic granular cytoplasm and variable amounts of collagenous stroma. Nuclei are generally small and centrally located (1). The infiltrative nature of these tumors, combined with prominent nucleoli, necessitates distinction from scirrhous carcinoma and apocrine carcinoma (13). The hallmark histologic feature aiding in differential diagnosis is the presence of granular cytoplasm within the tumor cells.

Immunohistochemically, BGCTs are negative for estrogen receptor, progesterone receptor, and human epidermal growth factor receptor 2. They are believed to originate from Schwann cells of neural origin, which explains their strong positivity for S100 protein, vimentin, and neuron-specific enolase (NSE), and negativity for pan-cytokeratin. Additionally, BGCTs often express CD68 and stain positive for periodic acid–Schiff (PAS), indicative of lysosomal activity in approximately 90% of cases (6). The Ki-67 proliferation index is an important marker of tumor aggressiveness (26). Given their typically benign nature, most of them exhibit a Ki-67 index of <2%. In evaluating potential malignancy, Ki-67 index must be considered in conjunction with clinical course and mitotic activity (26).

Malignant transformation is rare, accounting for <1% of all GCTs, including those of the breast (3). Nevertheless, cases of malignant BGCT have been reported. Malignant GCTs are classified as high-grade sarcomas, with high metastatic potential and poor prognosis (26). Features suggestive of malignancy include: tumor size ≥ 4 cm, increased mitotic rate (≥2 mitoses per 10 high-power fields at 400× magnification), rapid growth, evidence of local invasion, marked cellular pleomorphism (1).

Histopathologic confirmation should be obtained prior to treatment for suspected malignant lesions. Although diagnosis via fine-needle aspiration or intraoperative frozen section has been reported (12), this approach depends heavily on the expertise of the pathologist. Core needle biopsy (1, 3, 27), excisional biopsy (7, 16), and vacuum-assisted breast biopsy are considered more reliable methods. Histopathologic examination and immunohistochemistry remain the gold standard for diagnosis. Inappropriate pathological assessment may lead to overtreatment (2, 28, 29).

Complete surgical excision with negative margins remains the only treatment of choice (1). Wide local excision is the most widely accepted surgical strategy and is essential for further pathological evaluation after biopsy. Recurrence of benign BGCTs is extremely rare. Even in cases with positive margins, the risk of long-term recurrence is low (30). In the event of lymph node metastasis from malignant BGCTs, axillary lymph node dissection is warranted. Long-term follow-up (up to 10 years) is recommended (30).

This case is of particular interest due to the rarity of BGCTs in the breast. A thorough understanding of their clinicopathologic and radiologic features is critical for the accurate differentiation from breast carcinoma.

## Data Availability

The original contributions presented in the study are included in the article/supplementary material. Further inquiries can be directed to the corresponding authors.

## References

[1] De Simone N Aggon A Christy C. Granular cell tumor of the breast: clinical and pathologic characteristics of a rare case in a 14-year-old girl. J Clin Oncol. (2011) 29:e656–7. doi: 10.1200/JCO.2011.35.9448, PMID: 21646617

[2] Akbari A Behravan P Moradi A Akbari ME. Case report of a benign granular cell tumor resembling breast carcinoma in a young woman: A diagnostic challenge. Case Rep Oncol. (2024) 17:608–13. doi: 10.1159/000538771, PMID: 39015632 PMC11250677

[3] James NE Guan Y Musa F Cuffolo G. Granular cell tumour of the breast. BMJ Case Rep. (2024) 17(8):e258326. doi: 10.1136/bcr-2023-258326, PMID: 39153762

[4] Adeniran A Al-Ahmadie H Mahoney MC Robinson-Smith TM. Granular cell tumor of the breast: a series of 17 cases and review of the literature. Breast J. (2004) 10:528–31. doi: 10.1111/j.1075-122X.2004.21525.x, PMID: 15569210

[5] Miller JA Karcnik TJ Karimi S. Granular cell tumor of the breast: definitive diagnosis by sonographically guided percutaneous biopsy. J Clin Ultrasound. (2000) 28:89–93. doi: 10.1002/(SICI)1097-0096(200002)28:2<89::AID-JCU6>3.0.CO;2-N, PMID: 10641006

[6] Brown AC Audisio RA Regitnig P. Granular cell tumour of the breast. Surg Oncol. (2011) 20:97–105. doi: 10.1016/j.suronc.2009.12.001, PMID: 20074934

[7] Albasri AM Ansari IA Aljohani AR Alhujaily AS. Granular cell tumour of the breast in a young female: A case report and literature review. Niger J Clin Pract. (2019) 22:742–4. doi: 10.4103/njcp.njcp_282_18, PMID: 31089034

[8] Luo L Wang X Lin Y Ma X Tan A Chan R. Deep learning in breast cancer imaging: A decade of progress and future directions. IEEE Rev Biomed Engineering. (2025) 18:130–51. doi: 10.1109/RBME.2024.3357877, PMID: 38265911

[9] Kong X Zhang Q Wu X Zou T Duan J Song S. Advances in imaging in evaluating the efficacy of neoadjuvant chemotherapy for breast cancer. Front Oncol. (2022) 12:816297. doi: 10.3389/fonc.2022.816297, PMID: 35669440 PMC9163342

[10] Pötsch N Vatteroni G Clauser P Helbich TH Baltzer PAT. Contrast-enhanced mammography versus contrast-enhanced breast MRI: A systematic review and meta-analysis. Radiology. (2022) 305:94–103. doi: 10.1148/radiol.212530, PMID: 36154284

[11] van Nijnatten TJA Morscheid S Baltzer PAT Clauser P Alcantara R Kuhl CK. Contrast-enhanced breast imaging: Current status and future challenges. Eur J Radiol. (2024) 171:111312. doi: 10.1016/j.ejrad.2024.111312, PMID: 38237520

[12] El Aouni N Laurent I Terrier P Mansouri D Suciu V Delaloge S. Granular cell tumor of the breast. Diagn Cytopathol. (2007) 35:725–7. doi: 10.1002/dc.20736, PMID: 17924412

[13] Hammas N El Fatemi H Jayi S Hafid I Fikri G El Houari A. Granular cell tumor of the breast: a case report. J Med Case Rep. (2014) 8:465. doi: 10.1186/1752-1947-8-465, PMID: 25541096 PMC4307888

[14] Ito M Amari M Sato A Hikichi M. Granular cell tumor mimicking breast carcinoma: A report of two cases. Cureus. (2024) 16:e57500. doi: 10.7759/cureus.57500, PMID: 38707173 PMC11066709

[15] Leo C Briest S Pilch H Schütz A Horn LC Leinung S. Granular cell tumor of the breast mimicking breast cancer. Eur J Obstet Gynecol Reprod Biol. (2006) 127:268–70. doi: 10.1016/j.ejogrb.2006.01.026, PMID: 16849031

[16] Gogas J Markopoulos C Kouskos E Gogas H Mantas D Antonopoulou Z. Granular cell tumor of the breast: a rare lesion resembling breast cancer. Eur J Gynaecol Oncol. (2002) 23:333–4., PMID: 12214737

[17] Akatsu T Kobayashi H Uematsu S Tamagawa E Shinozaki H Kase K. Granular cell tumor of the breast preoperatively diagnosed by fine-needle aspiration cytology: report of a case. Surg Today. (2004) 34:760–3. doi: 10.1007/s00595-004-2784-7, PMID: 15338349

[18] Corines MJ Krystel-Whittemore M Murray M Mango V. Uncommon tumors and uncommon presentations of cancer in the breast. Curr Breast Cancer Rep. (2021) 13:331–46. doi: 10.1007/s12609-021-00435-x, PMID: 36589040 PMC9798716

[19] Irshad A Pope TL Ackerman SJ Panzegrau B. Characterization of sonographic and mammographic features of granular cell tumors of the breast and estimation of their incidence. J Ultrasound Med. (2008) 27:467–75. doi: 10.7863/jum.2008.27.3.467, PMID: 18314525

[20] Iglesias A Arias M Santiago P Rodríguez M Mañas J Saborido C. Benign breast lesions that simulate Malignancy: magnetic resonance imaging with radiologic-pathologic correlation. Curr Probl Diagn Radiol. (2007) 36:66–82. doi: 10.1067/j.cpradiol.2006.12.001, PMID: 17331838

[21] Abreu N Filipe J André S Marques JC. Granular cell tumor of the breast: correlations between imaging and pathology findings. Radiol Bras. (2020) 53:105–11. doi: 10.1590/0100-3984.2019.0056, PMID: 32336825 PMC7170582

[22] Scaranelo AM Bukhanov K Crystal P Mulligan AM O’Malley FP. Granular cell tumour of the breast: MRI findings and review of the literature. Br J Radiol. (2007) 80:970–4. doi: 10.1259/bjr/95130566, PMID: 17940129

[23] Aydin H Guner B Esen Bostanci I Bulut ZM Aribas BK Dogan L. Is there any relationship between adc values of diffusion-weighted imaging and the histopathological prognostic factors of invasive ductal carcinoma? Br J Radiol. (2018) 91:20170705. doi: 10.1259/bjr.20170705, PMID: 29299933 PMC5965983

[24] Moffa G Galati F Panzironi G Rizzo V Kripa E Pediconi F. Granular cell tumor of the breast: Tip and tricks on conventional and magnetic resonance imaging. Breast J. (2020). doi: 10.1111/tbj.14113, PMID: 33289271

[25] Zhu M Xu H Chen Y Peng Y. Multimodal ultrasonography findings of extramammary granular cell tumors: Two case reports. Front Oncol. (2023) 13:1136770. doi: 10.3389/fonc.2023.1136770, PMID: 37020870 PMC10067867

[26] Akahane K Kato K Ogiso S Sakaguchi K Hashimoto M Ishikawa A. Malignant granular cell tumor of the breast: case report and literature review. Breast Cancer. (2015) 22:317–23. doi: 10.1007/s12282-012-0362-1, PMID: 22467405

[27] Corso G Di Nubila B Ciccia A De Camilli E Vicini E Trentin C. Granular cell tumor of the breast: Molecular pathology and clinical management. Breast J. (2018) 24:778–82. doi: 10.1111/tbj.13036, PMID: 29900629

[28] Ohnishi H Nishihara K Tamae K Mitsuyama S Abe R Toyoshima S. Granular cell tumors of the breast: a report of two cases. Surg Today. (1996) 26:929–32. doi: 10.1007/BF00311799, PMID: 8931228

[29] Bauerfeind I Ditsch N Sittek H Diebold J. Reduction mammaplasty in granular cell tumour of the breast. Br J Plast Surg. (2004) 57:458–61. doi: 10.1016/j.bjps.2003.12.019, PMID: 15191830

[30] Papalas JA Wylie JD Dash RC. Recurrence risk and margin status in granular cell tumors of the breast: a clinicopathologic study of 13 patients. Arch Pathol Lab Med. (2011) 135:890–5. doi: 10.5858/2010-0430-OAR.1, PMID: 21732779

